# Mutual support between patients and family caregivers in palliative care: A qualitative study

**DOI:** 10.1177/02692163231205130

**Published:** 2023-10-13

**Authors:** Rachel McCauley, Karen Ryan, Regina McQuillan, Geraldine Foley

**Affiliations:** 1 Discipline of Occupational Therapy, School of Medicine, Trinity College Dublin, Dublin, Ireland; 2 St. Francis Hospice Dublin, Dublin, Ireland; 3 Mater Misericordiae University Hospital, Dublin, Ireland; 4 University College Dublin, Dublin, Ireland; 5 Beaumont Hospital, Dublin, Ireland; 6 Royal College of Surgeons in Ireland, Dublin, Ireland

**Keywords:** Family caregivers, patients, mutual support, reciprocity, psychosocial support, decision making, qualitative research, grounded theory

## Abstract

**Background:**

Patients in receipt of palliative care services are often viewed primarily as recipients of support from their family caregiver. There is a dearth of evidence in palliative care on what comprises mutual support between patients and their family caregivers in palliative care.

**Aim:**

To identify processes of mutual support between patients and family caregivers in palliative care.

**Design:**

Qualitative study comprising semi-structured interviews. Data were analysed using grounded theory procedures.

**Setting/participants:**

Fifteen patients with advanced illness (cancer *n* = 14, neurodegenerative *n* = 1) and 21 family caregivers recruited from a large regional-based hospice.

**Results:**

Mutual support between patients and family caregivers comprised two primary modes in which support was provided and received. Mutual support involved both patients and family caregivers providing similar types of support to each other, and which typically manifested as emotional support. However, mutual support also occurred when patients reciprocated by providing emotional support to their family caregivers to compensate for other forms of support which they felt no longer able to provide. Patients supported family caregivers by involving them in decision-making for care and both patient and family caregiver preferences were influenced by obligation to their respective other. Mutual support comprised both disclosure and concealment. Involving family caregivers in patient care decision-making was intended by patients to help family caregivers adjust to a caregiving role.

**Conclusions:**

The findings inform the development and delivery of psychosocial interventions for patients and family caregivers in palliative care aimed at facilitating supportive relations between them.

**What is already known about the topic?**

- The provision of support between patients and family caregivers in palliative care is often viewed as unidirectional (i.e. from caregiver to patient).
- There is a dearth of evidence focused on mutual support between patients and family caregivers in palliative care.

**What this paper adds?**

- Mutual support between patients and family caregivers in palliative care occurs when patients and family caregivers reciprocate in the provision of emotional care but also when patients are motivated to provide emotional support in return for everyday tasks they are no longer able to perform.
- Patient and family caregiver mutual obligation in palliative care can result in concealment of distress and care preferences.
- Patients in palliative care can seek to support family caregivers by involving them in the decision-making process for care.

**Implications for practice, theory or policy**

- Both open disclosure and concealment can feature as mutually supportive behaviours between patients and family caregivers in palliative care.
- Patient and family caregiver psychosocial interventions in palliative care need to account for how mutual support between patients and their family caregivers impacts on how both approach decision-making with respect to treatment and care.

## Background

In palliative care, family caregivers participate regularly in care provision for their ill family member.^1–3^ Family caregiving responsibilities in palliative care mean that family caregivers provide care directly to the patient, and in many cases, they operate as both a conduit and extension to formal support and care services.^4,5^ Family caregivers provide a combination of physical, psychological, emotional and financial support to the person they care for^6^ and can be involved in the decision-making process pertaining to care.^7^

The family caregiver in palliative care services is often conceptualised as *the* provider of support within the patient-family caregiver relationship.^8^ However, the caring relationship between people with life-limiting illness and their family caregivers can be bidirectional.^9,10^ People with life-limiting illness and their family caregivers have capacity to support one another by adapting collectively to advancing illness.^11^ Research on the dying person and family caregiver relationship has captured how mutual desire to remain steadfast in adversity is a component of caring.^12^ The burden that the patient feels that they place on their family caregivers and the responsibility that family caregivers feel towards their ill family member can result in patients and family caregivers prioritising each other’s needs over their own.^13,14^

Our previous work^15^ has identified few studies in palliative care which have focused on reciprocity in the provision of support between patients and family caregivers. A systematic review of limited evidence^15^ identified that patients with advancing conditions and family caregivers in palliative care can support one another by remaining positive for one another and by mutually adapting to the challenges of both living with life-limiting illness and negotiating palliative care. However, few studies have sought to examine specifically how patients receiving palliative care and their family caregivers support one another. In the Republic of Ireland, the focus of palliative care provision is for people to receive palliative care appropriate to their own needs, regardless of care setting or diagnosis. Our aim was to identify and explain how patients in receipt of palliative care and their family caregivers can be mutually supportive towards one another. Knowing how patients and family caregivers reciprocate in supporting one another is important because it can help sensitise healthcare professionals more fully to how patient and caregiver preferences are shaped by their perceived responsibility to one another.

## Methods

### Design

We conducted a qualitative study using a grounded theory approach.^16^ The grounded theory method is a systematic set of techniques and procedures for generating well conceptualised explanatory frameworks and even theory from data.^17^ It is well suited to investigating poorly understood phenomena. We used a grounded theory approach because we were focused on identifying key processes (i.e. actions and behaviours in response to context) that help explain the phenomenon of interest^16^ (in this case, mutual support between patients and family caregivers in palliative care). The Standards for Reporting Qualitative Research (SRQR)^18^ were referred to in the reporting of this study.

### Inclusion/exclusion criteria

Patient inclusion criteria for this study were that they had to be ⩾18 years old, have a formal diagnosis of a life-limiting condition(s) and be in receipt of palliative care. Inclusion criteria for family caregivers were that they were also ⩾18 years old and that they were recognised by a patient in receipt of palliative care as their primary family caregiver.

### Setting/participants

Between July 2021 and May 2022, 15 patients with advanced illness (cancer *n* = 14, neurodegenerative *n* = 1) and 21 family caregivers (total *n* = 36) were recruited from two hospice sites. The two hospice sites combined constitute a regional specialist palliative care service (inpatient, outpatient and home-based care) for a catchment area comprising a population of approximately 700,000 people. Specialist palliative care services at the sites were provided according to a national clinical programme model of care.^19^

The sample comprised 14 patient-family caregiver dyads (*n* = 28), seven family caregivers who participated without the patient they cared for, and one patient who participated without their respective family caregiver. Family caregivers participating on their own was for the most part due to acute deterioration in a patient’s health status between investigators’ approach and their scheduled interview. For the one patient who participated without their family caregiver, the caregiver had declined to participate because of their own distress. Family caregiver participants comprised spouses or partners (*n* = 10) and adult children (*n* = 11). The majority of patient participants (13 out of 15) were recruited from specialist community palliative care (i.e. specialist palliative care at home) and the remaining two patient participants were recruited from inpatient hospice. All patient participants were in advanced stages of their illness, and none had a prognosis of more than 18 months. The majority of the 15 patient participants (*n* = 9) had already ceased full, active treatment (e.g. radiation therapy, chemotherapy). Table 1 provides detail on the sample. Gatekeepers at each recruitment site assisted with recruitment. In the early stages of recruitment, the role of the gatekeepers was to inform eligible patients and caregivers about the study. As recruitment progressed, the gatekeepers were asked by the team to target eligible patients and caregivers that were best suited to further interrogate key concepts in the data.

**Table 1.** Summary of participants.

Participants	Condition of *patient participants*	Condition of *non-participating patients* cared for by *participating* family caregiver	Care setting at recruitment for patient and family caregiver participants	Participants’ gender and age	Patient and caregiver relationship
Total *N* = 36 (14 patient-caregiver dyads, 1 non-dyad patient participant and 7 non-dyad family caregiver participants) 8 dyads interviewed separately (16 interviews) 6 dyads interviewed together (6 interviews) 8 non-dyad participants (8 interviews)	*N* = 15 patient participants Metastatic cancer/advanced cancer (*n* = 14) Progressive multiple sclerosis (*n* = 1)	Metastatic cancer/advanced cancer (for 6 of the 7 non-dyad family caregiver participants) Cardiac failure (for 1 of the 7 non-dyad family caregiver participants)	*14 patient-family caregiver dyads* (SCPC for 12 patient-family caregiver dyads, and inpatient hospice for 2 patient-family caregiver dyads) *1 non-dyad patient participant*(SCPC) *7 non-dyad family caregiver participants* (SCPC for 6 non-dyad family caregiver participants, and outpatient hospice for 1 non-dyad family caregiver participant)	Men patients (*n* = 11) Women patients (*n* = 4) Men family caregivers (*n* = 4) Women family caregivers (*n* = 17) Patient age (40–90 years, median 65 years) Family caregiver age (22–80 years, median 52.5 years)	*Patient* Spouse/partner of family caregiver (*n* = 10) Parent of family caregiver (*n* = 5) Family caregiver Spouse/partner of patient (*n* = 10) Adult child of patient (*n* = 11)

### Data collection

As per grounded theory method, we analysed data during data collection to build concepts and categories, and to identify key processes in the data.^16^ Sampling was purposive to account for degrees of variation in participant situation and experience (e.g. variation in participants’ life stage, patient illness severity and patient-caregiver relationship). We also theoretically sampled for concepts in the data to expand and saturate key categories in the data.^16^ For example, when we identified that patients felt they were providing emotional support to their family caregiver, we (theoretically) sampled patients who had prior to the study, already drawn attention to the importance of emotional support between them and their family caregiver to identify their motivation to provide emotional support to their caregiver. In total, 30 semi-structured qualitative interviews were conducted with participants. Of the 14 patient-family caregiver dyads, 8 of these dyads were interviewed separately and 6 dyads requested to be interviewed together (i.e. both patient and family caregiver in one interview). The family caregivers and the patient who participated without their respective other were each interviewed alone. All patient participants were in their recruitment site (i.e. at home or as an inpatient) when interviewed. Interview schedules were formulated with the purpose of generating data in the context of the research aim, a recent systematic review of the evidence already conducted^15^ and wider literature. Open-ended interview questions asked of participants pertaining to mutual support are detailed in the online Supplemental Material.

R.McC conducted all interviews. The average duration of interviews was 43 min. Due to restrictions imposed by COVID-19 during data collection, 27 of the 30 interviews were conducted remotely and the remainder in person. Of those interviews conducted remotely, 25 of these interviews were conducted by phone (*n* = 29) and 2 conducted via the video-conferencing platform zoom^20^ (*n* = 3). All interviews were digitally audio-recorded and each interview was transcribed by R.McC. Field notes were also compiled by R.McC after each interview to help contextualise interviews for analysis. Table 1 includes a summary of the interviews conducted. Interview transcripts were returned to all but four participants who requested that their transcript not be returned to them. Member checking was also performed during interviews to confirm participants’ responses.^21^ No participant requested adjustment to or deletion of their data.

### Data analysis

The data were analysed through grounded theory coding procedures.^16^ First, the (transcript) data were coded to identify key incidents and behaviours indicating potential characteristics of mutual support. Through constant comparison of the data,^16^ these codes were interrogated further and expanded to build concepts and categories (larger concepts), make tentative connections between concepts and categories, and account for both similarities and differences in the data.^16^ The final stages of the analysis focused on refining key categories and the relationship between them to identify key processes that explained how patients and family caregivers sought to be mutually supportive to their respective other. For instance, following on from the example of early theoretical sampling mentioned in data collection, we identified that patients’ desire to provide emotional support was motivated in part by the loss of other forms of support they felt able to provide. At the same time, family caregivers had already reported feeling responsible for the patient and for many caregivers, responsible care included being of emotional support to the patient. We then probed and prompted both patients and caregivers as to why *reciprocation* of emotional support was of benefit to both the patient and caregiver. Subsequent analysis of the data revealed that mutual emotional support was necessary for patients and caregivers to better understand each other’s viewpoint and balance each other’s needs against their own. R.McC conducted the analysis which was cross-checked by G.F who also assisted with interpretation of the findings. Cross-checking and interpretation of the data was aided by detailed memoing of the data^22^ by R.McC. NVivo12 qualitative data analysis software was used as tool to assist with coding the data. An example of category identification is available in the Supplemental Material.

### Ethics

Ethical approval to conduct this study was granted by the Faculty of Health Sciences Research Ethics Committee, Trinity College Dublin (ref 191002) and by the Research Ethics Committee of St. Francis Hospice Dublin. Informed written consent was provided by participants.

## Findings

Here, we report from our study on how both patients and family caregivers provided support to their respective other. We report on our primary categories which explained processes of mutual support between patients and family caregivers: Mutual support manifesting as emotional support; reciprocation by different modes of support; and care decision-making in the context of mutual support.

### Mutual support manifesting as emotional support

Mutual support comprised both patients and family caregivers reciprocating in the form of emotional support. Patients and family caregivers reported engaging in expressions of affection and concern. In many cases, this resulted in open and mutual disclosure focused on reassuring their respective other:

She’s [patient] very. . . she’s very thankful to me. She can’t say enough ‘What would I do without you?’ and ‘I’d be lost without you’ and then I tell her I love her an awful lot, and she registers. . . that registers with her, [she says] ‘I love you as well’ kind of thing. (FCG 17)

She [family caregiver] would go quiet and there do be things on her mind. So we just talk about it. I try to just put her mind at ease and everything like that. Sometimes I chisel away at her, and she breaks down and tells me, and then we can open up. . . as I say a problem shared you know. (Patient 7)

Attempting to remain positive for one another and seeking to maintain normalcy or constancy in the face of change underpinned how most participants sought to provide emotional support to their respective other. Patient 5 and his family caregiver shared:

If you haven’t got a positive attitude, [if] you’ve got a negative attitude, it’s going to cause problems for them. (Patient 5)

Trying to be as normal. . . as normal as you were before. . . Going for walks or going out for a meal, or the usual things that we would have done before he [patient] got the diagnosis. . . to see that we are still the same as such. (FCG 5)

Patients and family caregivers also supported their respective other emotionally by wanting to understand each other’s viewpoint on matters that affected both the patient and family caregiver. Indeed, participants indicated that having a shared understanding of their respective other enabled them to cope with the challenges they faced:

I think we are an emotional support for each other because we understand each other. You know we say we are singing from the same hymn sheet, and we are pretty I think understanding of one another, and the way each other handles things and copes with things. (Patient 3)

Overall, providing emotional support was understood collectively by participants as ‘being there’ for one another. Despite the emotional strain of living with life-limiting illness, both patients and family caregivers recognised the importance of being emotionally available to the other in time of need:

The day-to-day stuff. . . being there for one another is probably the most important part I think of how we are dealing with things at the moment. (FCG 1)

There are certain things that I can do to help the situation with [family caregiver]. We sort of marry one another in that respect, you know, I give [family caregiver] emotional help in whatever way and again, she gives it to me a hundred-fold. (Patient 10)

### Reciprocation by different modes of support

In the context of patient participants’ deteriorating health, patients had already become dependent to varying degrees on their family caregiver for personal and instrumental activities of daily living. Whilst family caregivers perceived themselves as remaining able to provide a broad spectrum of support (i.e. physical, psychological and social) and at key points along the patient illness trajectory, patients perceived that they became increasingly limited in the degree and type of support that they could provide – particularly for activities that required high degrees of physical and/or mental exertion. Patient 1 explained:

I would have always held the house down, did the washing, the cooking, the cleaning the shopping, and I would have always done all that. And that has changed drastically. I don’t do any of that anymore. I’ve been told I can’t lift anything heavier than a kettle. . . So [family caregiver] role and my daughter’s role as well. . . they have taken on that role that was mine.

Nonetheless, patients recognised the strain that their family caregivers were experiencing and continued to reciprocate to the best of their ability to cater for the psychological and emotional needs of their family caregiver. Overt expressions of emotional support from patients were appreciated by family caregivers:

When she [patient] just grabs your hand and says, ‘I love you and I know I'm putting you under stress. I’m sorry’. . . It means the world to me. (FCG 4)

Even though she’s [patient] sick, dying. . . she’s still trying to help you work through your stupid, little problems that you have because she knows that they are big problems to me. She still makes sure that you’re okay, that you can pay your mortgage, and is your car okay and are the children and everything fine. (FCG 20)

However, patient reciprocation through different modes of support not only involved overt affection and open disclosure. It could also result (conversely) in patient concealment of their distress and in taking actions aimed at alleviating the burden of care encountered by their family caregiver in a family caregiving role:

It’s a bit hard because I’m going on without them. . . I don’t think there’s really any point in making things worse for [family caregiver] than there is if you like, so I deal with it [illness] myself. (Patient 5)

They are [family caregiver] not looking after me if I became incontinent or paralysed. . . I wouldn’t be able to cope even thinking about it. I’m nearly breaking out in a sweat like thinking about it, so no there’s not a chance I’d let them look after me like that. (Patient 6)

In some cases, concealment of both physical and emotional distress was maintained by patients up until the point at which they could no longer conceal their distress to their family caregivers. Patient 6 above also shared:

Normally, I’d always be the one to put up ‘Oh I’m fine!’, I’d always put a front on. But then lately I don’t. . . I can’t do it anymore. Physically don’t have the energy to hide it anymore.

Overall, we found that patients were particularly sensitive to family caregivers’ own self-care and frequently encouraged family caregivers where possible to engage in activities outside of their caregiving role.

### Care decision-making in the context of mutual support

Both patients and family caregivers across the sample were cognisant of the fact that any decisions made by the patient in relation to palliative care (either alone or together with their family caregiver), impacted both the patient and family caregiver. The majority of patients (*n* = 12) involved family caregivers in discussion about their care particularly when confronted with decisions about their care (e.g. symptom control, location of care, planning of future care, etc.) and this was intended as means of support to their family caregiver. Patient and family caregiver preferences for patient care were modulated by a sense of obligation they felt to their respective other. In the case of patients, this included feeling a need to live on to be with their family caregivers but also for others, not wanting to extend burden on family caregivers. For example, patient 7 and patient 13 communicated:

If I had a choice and offered the more aggressive treatment and spend more time with [family caregiver], absolutely [I would] take more aggressive treatment. (Patient 7)

I would have to think of [family caregiver]. You couldn’t just be greedy and grab on to them [treatments] without consulting them you know. Because it would affect [family caregiver] in the long run. (Patient 13)

For family caregivers, obligation included alleviating any pressure on patients to live on in distress including in some cases, concealing what they themselves wished for the patient’s care:

I’m trying to be unselfish and it would be difficult to let her go. . . but I think if it [treatment] was going to prolong her life in a very uncomfortable way, I wouldn’t go there. . . as hard as it would be to let her go. (FCG 11)

The intention behind involving family caregivers in decision-making was to assist family caregivers adjust to rapidly changing circumstances, particularly in cases where the patient felt that the family caregiver was struggling to come to terms with the patient’s prognosis. As patient 15 stated:

We make them [decisions] together. . . For example, I decided to come off the chemo after eight months, and I took a break away from it for about two months. Then decided to try the new treatment that they were giving because she [family caregiver] doesn’t want me to die. That was decision made from me and [family caregiver].

Family caregivers being involved in decisions about care or simply in discussion about care could result in bringing the family caregiver and patient closer together in their relationship:

We talk about everything now. . . He [patient] always talks about what he wants to do and his decisions and stuff like that. He gets my. . . opinions on stuff and we decide as a family. . . decisions and talking about things. . . I think we have even become closer since the diagnosis. (FCG 7)

However, involvement in decision-making also rendered feelings of control for the family caregiver in their caregiving role and in some cases, made family caregivers feel more in control of the patient’s care:

The more I can get through [deciding] the situation, then I can control the situation. . . I couldn’t in all honesty give his [patient] care over now to somebody else. (FCG 9)

## Discussion

### Main findings

We have identified that mutual support between patients and family caregivers in palliative care can involve two different modes of reciprocation. First, mutual support involved both patients and family caregivers providing emotional support; it was notable that patients and caregivers often provided emotional support in ways that were similar or mirrored the way that the other person provided support. Second, mutual support occurred when patients reciprocated by providing emotional support in recognition of caregiver strain and to compensate for other forms of support which patients felt they were no longer able to render to their family caregiver. Reciprocation in emotional support comprised actions and behaviours which were clearly overt to both patients and family caregivers (e.g. open disclosure and explicit expression of affection) but also actions and behaviours intended to conceal (e.g. concealment of distress) and/or which could result in concealment (e.g. constraint in overtly expressing preferences), out of obligation to their respective other. Patient involvement of family caregivers in decision-making was underpinned by their need to be of support to their family caregiver and to assist them in their role as a caregiver, which in turn led family caregivers to feel more in control of the challenges surrounding caregiving and patient care.

### What this study adds

Our findings resonate with and support existing evidence on supportive relations between patients and family caregivers in palliative care. The benefit of mutual affection,^10^ open disclosure,^23^ positivity and seeking normalcy^24^ and of overt expression of support,^25^ have been documented and found to assist both patients and family caregivers cope with challenging circumstances in life-limiting illness. In our study, open communication between patients and family caregivers was also a basis for mutual understanding.^23^ Our findings are evidence that patients and family caregivers have capacity to support one another during periods of advanced illness and distress.^15^ Patients with severe illness in palliative care can still reciprocate in a caring role^26^ and remain sensitive to the competing needs of their family caregiver.^27^ Importantly, *both* patient and family caregiver preferences for patient care are shaped by feelings of obligation towards each other.^12,13^

Our findings pertaining to obligation corroborate existing evidence that caring between patients and family caregivers in palliative care can be mutual^28^ and that both patients and family caregivers can feel constrained in their own preferences out of obligation to one another.^29^ However, our findings are also novel because we identified that mutual obligation features even when patients feel unable to reciprocate in specific forms of supportive care. Moreover, although a sense of obligation by patients resulted in taking actions intended to alleviate caregiver burden,^30^ patients still involved their family caregivers in decision-making about patient care. Our findings pertaining to patient and family caregiver concealment also extend our understanding of patient and family caregiver relational decision-making in palliative care. To add to published evidence,^15^ mutual concealment is not only an antecedent to or consequence of mutual conflict. It also manifests when patients and family caregivers attempt to minimise distress for their respective other. Finally, it is already known that family caregivers feel they lose control when unsupported by professional services.^31^ We have identified that family caregivers can gain control when the patient involves them in their care.

### Implications of findings for clinical practice and research

The findings of our study have implications for both clinical practice and research. The study was designed to identify mutually supportive behaviours between patients and family caregivers in palliative care. We knew from our previous work in the field^15,29^ that patients and family caregivers in palliative care can seek to support one another. However, in this study, we identified behaviours of both patients and family caregivers (e.g. concealment, constraint) intended to be supportive are behaviours that are ordinarily perceived by healthcare professionals and researchers as maladaptive.^32^ Moreover, although feelings of obligation underpinned mutual support, it also resulted in patients and family caregivers feeling constrained in their choice of preferences for care. Research focused on delineating more clearly between patient and family caregiver mutually supportive behaviours in the decision-making process for care and mutually supportive behaviours that can manifest and operate outside of that process, is warranted. Such studies would help inform the development and delivery of psychosocial interventions for patients and family caregivers in palliative care when the focus of the intervention is also on helping patients and family caregivers engage with palliative care services.^33,34^

## Strengths and limitations

Our study has both strengths and limitations. Despite the difficulties imposed by COVID-19, we managed to recruit in specialist palliative care both patients with advanced illness and family caregivers for a qualitative study where the focus was on relations between patients and family caregivers. In doing so, we have explained how the patient and family caregiver in palliative care can be supportive of one other. Although our findings are not generalisable to the wider population of patients with advanced illness and family caregivers in palliative care, they do allow for comparison across both similar and different contexts.^16^

All but one of the patient participants were cancer patients and so we did not capture a spectrum of patient diagnoses beyond cancer. In addition, a small proportion of the sample (*n* = 8) did not participate as dyads. Hence, in these cases, data generated were less contextualised to their respective other. Our initial plan had been to conduct in-person interviews with all participants. However, due to COVID-19, we had to change our mode of data collection in most cases to remote interviewing and primarily to phone interviews as many participants did not feel readily able to engage with videoconferencing platforms. More traditional in-person interviews or video-based online interviews instead of phone interviews could have allowed the interviewer to account more effectively for participant demeanour in interviews.^35,36^ Finally, recruitment was limited to one (albeit large) regional-based hospice service and largely to specialist community palliative care. Our findings are contextualised to participants’ experiences of specialist palliative care services in that region and for the most part to specialist community palliative care.

## Conclusions

Our study is one of a limited number of qualitative studies in palliative care which has reported on mutual support between patients and family caregivers.^15,24^ We recruited participants through specialist palliative care services and so our findings might not be as transferrable to non-specialist palliative care settings. Nonetheless, we have explained key processes of mutual support between patients and family caregivers, intended to assist healthcare professionals in palliative care who are typically tasked with supporting the needs of (and facilitating decision-making for) both patients and family caregivers. The findings of the study are very relevant for patient and family caregiver psychosocial interventions in palliative care where the emphasis is on effective communication and maintaining relationships, particularly where healthcare professionals need to help patients and family caregivers understand their choices in relation to each other and within the possibilities and constraints of their situation. The findings also serve as the basis for developing dyadic interventions focused specifically on mutual support between patients and family caregivers to improve patient and caregiver relatedness in specialist palliative care.

## Supplemental Material

sj-pdf-1-pmj-10.1177_02692163231205130 – Supplemental material for Mutual support between patients and family caregivers in palliative care: A qualitative study Click here for additional data file. Supplemental material, sj-pdf-1-pmj-10.1177_02692163231205130 for Mutual support between patients and family caregivers in palliative care: A qualitative study by Rachel McCauley, Karen Ryan, Regina McQuillan and Geraldine Foley in Palliative Medicine

sj-pdf-2-pmj-10.1177_02692163231205130 – Supplemental material for Mutual support between patients and family caregivers in palliative care: A qualitative study Click here for additional data file. Supplemental material, sj-pdf-2-pmj-10.1177_02692163231205130 for Mutual support between patients and family caregivers in palliative care: A qualitative study by Rachel McCauley, Karen Ryan, Regina McQuillan and Geraldine Foley in Palliative Medicine
