# Supplementary material for: Mutual support between patients and family caregivers in palliative care: A qualitative study
Source: Palliat Med. 2023 Oct 13;37(10):1520–8. doi: 10.1177/02692163231205130 (PMC10657498; doi:10.1177/02692163231205130)
Supplement: sj-pdf-1-pmj-10.1177_02692163231205130 – Supplemental material for Mutual support between patients and family caregivers in palliative care: A qualitative study [file sj-pdf-1-pmj-10.1177_02692163231205130.pdf]

Supplementary File (Interview schedule)

Open-ended questions on mutual support for patient

- What are your experiences so far of palliative care with [family caregiver]?
- How do you think both of you are coping?
- What do you think is the best kind of help or support to get from [family caregiver]? What makes it difficult or easy to accept support from [family caregiver]?
- How would you describe the way you rely (or might not rely) on [family caregiver]?
- In what way(s) do you feel you are a support (or not) to [family caregiver]?
- What do you think is the best kind of help or support that you give or can give to [family caregiver]?
- How might what you want for your care (e.g., medical treatments, multi-disciplinary care, home-based care, psychological support) be influenced by what you think is best for your [family caregiver]?
- How might your responsibility to [family caregiver] impact your preference for care and how you decide about your care?
- Is there anything else you would like to say that you have not had an opportunity to say?

Open-ended questions on mutual support for family caregiver

- What are your experiences so far of palliative care with [patient]?
- How do you think both of you are coping?
- In what way(s) do you feel you are a support (or not) to [patient]?
- What do you think is the best kind of help or support that you give or can give to [patient]? What makes it difficult or easy to provide help to [patient]?
- What do you think is the best kind of help or support that [patient] provides or can provide to you?
- How would you describe the way you rely (or might not rely) on [patient]?
- How might what you want for [patient's] care (e.g., medical treatments, multi-disciplinary care, home-based care, psychological support) and for your own care (e.g., any type of help), be influenced by what you think is best for [patient]?
- How might your responsibility to [patient] impact your preferences for care?

- Is there anything else you would like to say that you have not had an opportunity to say?
