# Supplementary material for: Mutual support between patients and family caregivers in palliative care: A qualitative study
Source: Palliat Med. 2023 Oct 13;37(10):1520–8. doi: 10.1177/02692163231205130 (PMC10657498; doi:10.1177/02692163231205130)

Category – ‘Mutual support manifesting as emotional support’

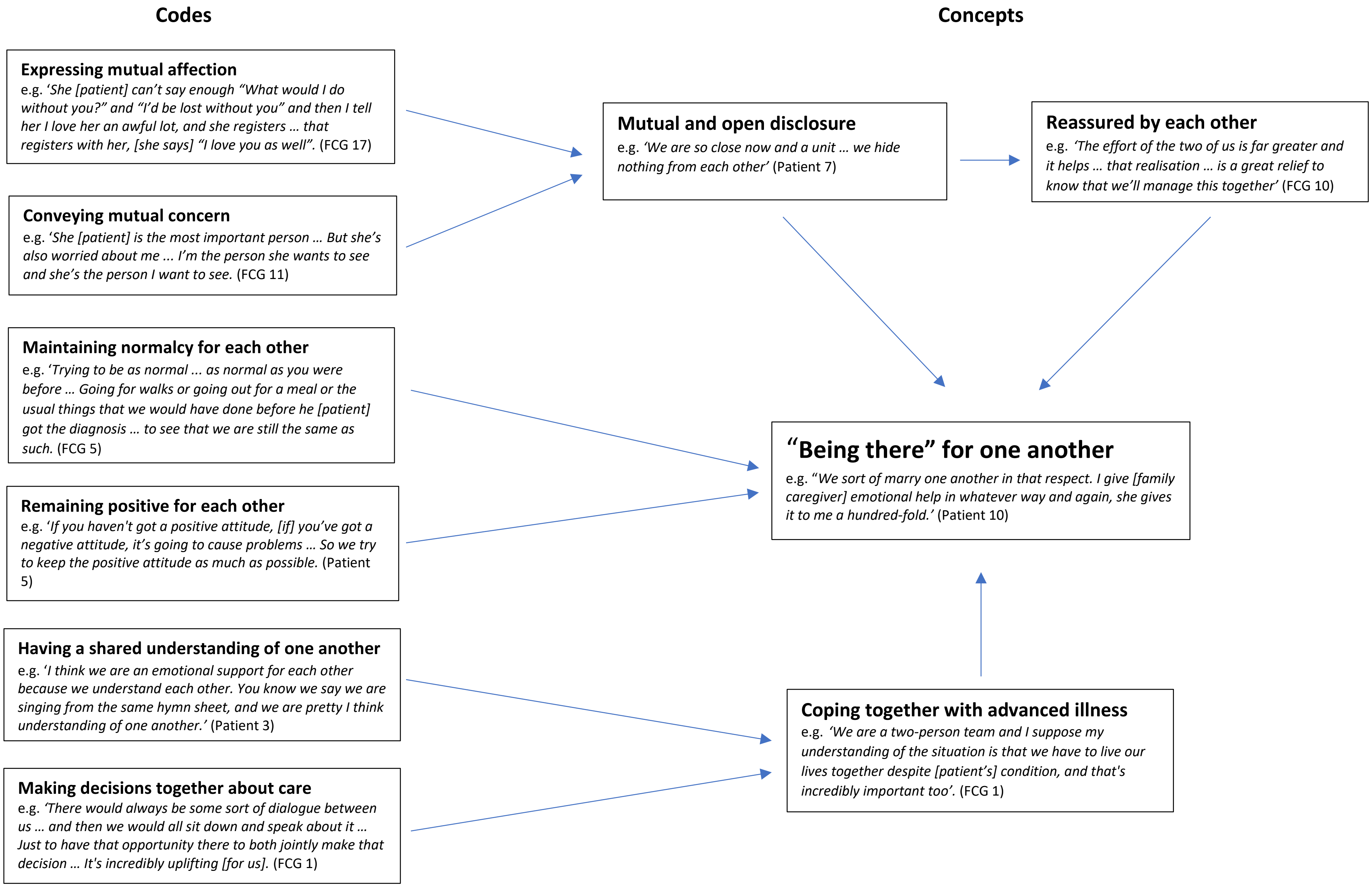

Supplement: sj-pdf-2-pmj-10.1177_02692163231205130 – Supplemental material for Mutual support between patients and family caregivers in palliative care: A qualitative study [file sj-pdf-2-pmj-10.1177_02692163231205130.pdf]
